# Effects of immune-mediated inflammatory diseases on cardiovascular diseases in patients with type 2 diabetes: a nationwide population-based study

**DOI:** 10.1038/s41598-022-15436-8

**Published:** 2022-07-07

**Authors:** Oh Chan Kwon, Kyungdo Han, Jaeyoung Chun, Ryul Kim, Seung Wook Hong, Jie-Hyun Kim, Young Hoon Youn, Hyojin Park, Min-Chan Park, Gastroenterology, Neurology and Rheumatology National Data Science Research (GUARANTEE) Group, Oh Chan Kwon, Kyungdo Han, Jaeyoung Chun, Ryul Kim, Seung Wook Hong, Jie-Hyun Kim, Young Hoon Youn, Hyojin Park, Min-Chan Park

**Affiliations:** 1 Division of Rheumatology, Department of Internal Medicine, Yonsei University College of Medicine, Seoul, Korea; 2 Department of Statistics and Actuarial Science, Soongsil University, 369 Sangdo-ro, Dongjak-gu, Seoul, 06978 Korea; 3 Division of Gastroenterology, Department of Internal Medicine, Yonsei University College of Medicine, 20, Eonju-ro 63-gil, Gangnam-gu, Seoul, 06229 Korea; 4 Department of Neurology, Inha University Hospital, Incheon, Korea; 5 Department of Gastroenterology, University of Ulsan College of Medicine, Asan Medical Center, Seoul, Korea

**Keywords:** Medical research, Outcomes research, Type 2 diabetes

## Abstract

Both type 2 diabetes and immune-mediated inflammatory diseases (IMIDs), such as Crohn’s disease (CD), ulcerative colitis, rheumatoid arthritis (RA), ankylosing spondylitis (AS), and psoriasis (PsO) are risk factors of cardiovascular disease. Whether presence of IMIDs in patients with type 2 diabetes increases their cardiovascular risk remains unclear. We aimed to investigate the risk of cardiovascular morbidity and mortality in patients with type 2 diabetes and IMIDs. Patients with type 2 diabetes without cardiovascular disease were retrospectively enrolled from nationwide data provided by the Korean National Health Insurance Service. The primary outcome was cardiovascular mortality, and the secondary outcomes were myocardial infarction (MI), stroke, and all-cause mortality. Inverse probability of treatment weighting (IPTW)-adjusted Cox proportional hazard regression analysis was performed to estimate the hazard ratios (HRs) and 95% confidence intervals (95% CIs) for each IMID. Overall 2,263,853 patients with type 2 diabetes were analyzed. CD was associated with a significantly higher risk of stroke (IPTW-adjusted HR: 1.877 [95%CI 1.046, 3.367]). UC was associated with a significantly higher risk of MI (1.462 [1.051, 2.032]). RA was associated with a significantly higher risk of cardiovascular mortality (2.156 [1.769, 2.627]), MI (1.958 [1.683, 2.278]), stroke (1.605 [1.396, 1.845]), and all-cause mortality (2.013 [1.849, 2.192]). AS was associated with a significantly higher risk of MI (1.624 [1.164, 2.266]), stroke (2.266 [1.782, 2.882]), and all-cause mortality (1.344 [1.089, 1.658]). PsO was associated with a significantly higher risk of MI (1.146 [1.055, 1.246]), stroke (1.123 [1.046, 1.205]) and all-cause mortality (1.115 [1.062, 1.171]). In patients with type 2 diabetes, concomitant IMIDs increase the risk of cardiovascular morbidity and mortality. Vigilant surveillance for cardiovascular disease is needed in patients with type 2 diabetes and IMIDs.

## Introduction

Type 2 diabetes, a metabolic disease with an increasing global prevalence, is associated with an increased risk of cardiovascular disease^1–3^. Importantly, cardiovascular disease is a major cause of increased mortality in patients with type 2 diabetes^4,5^. Accurate cardiovascular risk stratification in these patients may result in stricter monitoring and better outcomes in high-risk patients^6^.

Immune-mediated inflammatory diseases (IMIDs) are a heterogeneous group of chronic inflammatory diseases that affect the inner barrier (gut and joints) and/or outer barrier (skin) of the body^7^. Crohn’s disease (CD) and ulcerative colitis (UC) are IMIDs that mainly affect the gut, rheumatoid arthritis (RA) and ankylosing spondylitis (AS) are IMIDs that mainly affect the joints, and psoriasis (PsO) is an IMID that mainly affects the skin^8–12^. A large body of evidence suggests that IMIDs are associated with an increased risk of cardiovascular disease^13–16^. A population-based study has demonstrated that inflammatory bowel disease (IBD, i.e., CD and UC) confers a higher risk of myocardial infarction (MI) (odds ratio [OR] 1.25)^13^. Similarly, a higher risk of cardiovascular disease has also been reported in IMIDs that mainly affect the joints; the reported pooled relative risk for cardiovascular disease was 1.48 in patients with RA^14^, and the OR for MI was 1.6 in patients with AS^15^. A higher risk of MI (OR 1.25) was also observed in patients with PsO^16^.

Although IMIDs are well-known to increase the risk of cardiovascular disease in the general population^13–16^, it remains unclear whether the presence of IMIDs also increases the risk of cardiovascular disease in patients with type 2 diabetes who are already at a higher risk of cardiovascular disease. A comprehensive understanding of the influence of IMIDs on the cardiovascular disease in patients with type 2 diabetes can aid in better cardiovascular risk stratification in these patients. Considering that the prevalence of IMIDs is relatively low (approximately 3%)^5^, a population-based study is needed to sufficiently assess the influence of IMIDs on the risk of cardiovascular disease in patients with type 2 diabetes. In this study, we used nationwide population data to evaluate the risk of cardiovascular morbidity and mortality in patients with type 2 diabetes and IMIDs.

## Methods

### Data source

The nationwide population data from the Korean National Health Insurance Service (NHIS) claims database were used in this study. The NHIS provides comprehensive data that includes the demographics, socioeconomic status, medical treatments and procedures, disease diagnoses according to the International Classification of Diseases, Tenth Revision (ICD-10), and rare intractable disease (RID) registration information^17^. In the Korean RID system, a diagnosis is based on the uniform diagnostic criteria provided by the NHI and is carefully reviewed by the corresponding healthcare institution as well as the NHI before registration. The profile of the data source has been described previously^18^. All participants registered in the NHIS database are advised to undergo national health check-ups every 2 years. The categories of health check-up data include the anthropometric data, blood pressure, and laboratory data, such as serum fasting glucose levels, cholesterol levels, and creatinine levels. Using standardized self-reporting questionnaires, data regarding previous medical history and lifestyle factors, including smoking, alcohol consumption, and physical activity, were collected. This study was approved by the Institutional Review Board (IRB) of Gangnam Severance Hospital (IRB No: 3-2020-0269). Owing to the retrospective nature of this study, the requirement for informed consent was waived and approved by the IRB of Gangnam Severance Hospital. The study was performed in accordance with the Declaration of Helsinki, the relevant guidelines and regulations.

### Study cohort

**Figure 1:**
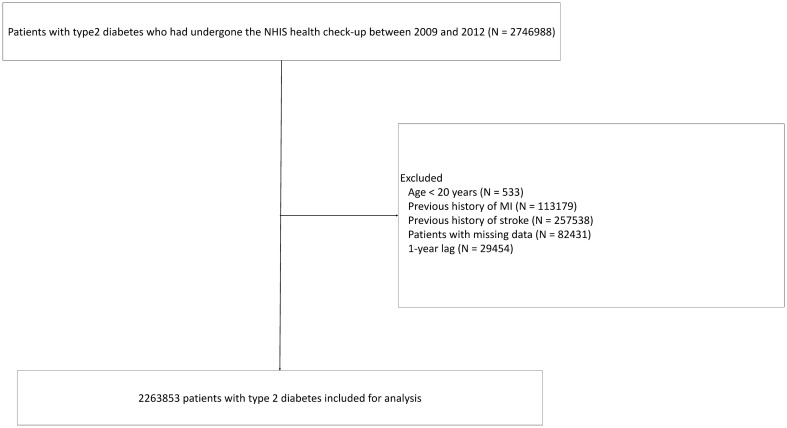
Flowchart depicting inclusion of patients. NHIS, National Health Insurance Service; MI, myocardial infarction.

We initially selected 2,746,988 patients with type 2 diabetes who underwent the NHIS health check-up between January 1, 2009 and December 31, 2012. Patients with type 2 diabetes were defined as those with ICD-10 codes E11–14 and at least one annual claim of a prescription of anti-diabetic medications^19^. The following patients were excluded (Fig. 1): (i) younger than 20 years (n = 533); (ii) previous history of MI (n = 113,179); (iii) previous history of stroke (n = 257,538); (iv) missing data regarding any of the following: regular physical activity (n = 1364), height (n = 713), weight (1160), body mass index (n = 1207), systolic blood pressure (n = 677), diastolic blood pressure (n = 683), proteinuria (n = 13,511), hemoglobin (n = 688), fasting glucose (n = 481), total cholesterol (n = 464), aspartate aminotransferase (n = 474), alanine aminotransferase (n = 504), gamma-glutamyl transferase (n = 488), waist circumference (n = 977), triglyceride (n = 573), high density lipoprotein cholesterol (n = 624), low density lipoprotein cholesterol (n = 31,339), creatinine (n = 517), smoking (n = 8600), and alcohol consumption (n = 23,876); and (v) MI, stroke, or death occurred within 1 year from the baseline (i.e., the date of the first health check-up between January 1, 2009 and December 31, 2012) (n = 29,454). The remaining 2,263,853 patients with type 2 diabetes were included in the analysis (Fig. 1).

Patients were classified according to the presence of each IMID (CD, UC, RA, AS, and PsO). The prevalence of each IMID and its effect on cardiovascular morbidity and mortality were assessed. Patients were followed up from the baseline till the date of a cardiovascular event, death, or December 31, 2020, whichever was earlier.

### Definition of IMIDs, comorbidities, and study outcomes

CD was defined as ICD-10 code K50 with RID code V130^20,21^; UC was defined as ICD-10 code K51 with RID code V131^20,21^; RA was defined as ICD-10 code M05 with RID code V223; AS was defined as ICD-10 code M45 with RID code V140^22^; and PsO was defined as ICD-10 code L40^23^. Baseline comorbidities were defined as follows: hypertension was defined as ICD-10 codes I10‒I13 and I15 with prescriptions for antihypertensive agents or systolic or diastolic blood pressure ≥ 140 mmHg or ≥ 90 mmHg, respectively^24^; and dyslipidemia was defined as ICD-10 code E78 with prescriptions for lipid-lowering agents or serum total cholesterol ≥ 240 mg/dL^24^. The primary outcome was cardiovascular mortality (death due to MI and/or stroke). The secondary outcomes included the incidence of MI and stroke, and all-cause mortality. MI was defined as ICD-10 codes I21 or I22 during hospitalization or these codes being recorded at least twice during the study period^19^. Stroke was defined as ICD-10 codes I63 or I64 during hospitalization with claims for brain magnetic resonance imaging or computed tomography^19^.

### Statistical analysis

Continuous variables of parametric and non-parametric distributions are expressed as mean (± standard deviation) and median (interquartile range), respectively. Categorical variables are expressed as numbers (%). Continuous variables were compared using independent Student’s *t* test or Mann–Whitney U test. Categorical variables were compared using χ2 test. The incidence of outcomes was expressed as the number of events per 1000 person-years. The cumulative incidences of outcomes were analyzed using the Kaplan–Meier method and compared using log-rank test according to the presence of each IMID. Hazard ratios (HRs) and the corresponding 95% confidence intervals (CI) for the outcomes in each IMID were estimated using Cox proportional hazard models. Model 1 was adjusted for none of the covariates (univariable analysis). Multivariable models were first adjusted for age and sex (model 2) and additional adjustments for other confounders in the following models (models 3–6). Bonferroni correction was performed for multiple comparisons. To balance the baseline characteristics between patients with and without each IMID, an inverse probability of treatment weighting (IPTW)-adjusted model was used. The propensity score for each IMID used in the IPTW-adjusted model was estimated by multiple logistic regression analysis including all variables in Table 1. All p-values were two-sided, and a *p*-value < 0.05 was considered statistically significant. Statistical analyses were performed using SAS version 9.4 (SAS Institute, Cary, NC, USA), and Stata statistical software v12 (StataCorp, College Station, TX, USA).

**Table 1:** Baseline characteristics of patients with type 2 diabetes according to the presence of immune-mediated inflammatory diseases.

	Total population	No CD	CD	*p*	No UC	UC	*p*	No RA	RA	*p*	No AS	AS	*p*	No PsO	PsO	*p*
Number of patients	2,263,853	2,263,692	161		2,262,964	889		2,259,950	3903		2,263,050	803		2,244,425	19,428	
Male sex, n (%)	1,388,001 (61.31)	1,387,888 (61.31)	113 (70.19)	0.0208	1,387,380 (61.31)	621 (69.85)	< .0001	1,386,986 (61.37)	1015 (26.01)	< .0001	1,387,327 (61.3)	674 (83.94)	< .0001	1,374,944 (61.26)	13,057 (67.21)	< .0001
Age, years, Mean ± SD	55.65 ± 12.31	55.65 ± 12.31	51.39 ± 13.43	< .0001	55.65 ± 12.31	56.61 ± 12.15	0.0204	55.64 ± 12.32	61.71 ± 9.27	< .0001	55.65 ± 12.31	50.05 ± 12.64	< .0001	55.64 ± 12.32	57.37 ± 11.72	< .0001
BMI, kg/m^2^, Mean ± SD	25.07 ± 3.69	25.07 ± 3.69	23.53 ± 3.45	< .0001	25.07 ± 3.69	24.38 ± 3.03	< .0001	25.07 ± 3.69	23.76 ± 3.45	< .0001	25.07 ± 3.69	25.33 ± 3.57	0.0427	25.07 ± 3.7	25.07 ± 3.41	0.745
Waist circumference, cm, Mean ± SD	85.21 ± 8.85	85.21 ± 8.85	82.15 ± 8.83	< .0001	85.21 ± 8.85	84.4 ± 8.06	0.0065	85.22 ± 8.85	81.83 ± 8.91	< .0001	85.21 ± 8.85	86.94 ± 9.15	< .0001	85.2 ± 8.85	86.21 ± 8.63	< .0001
Smoking, n (%)				0.2004			< .0001			< .0001			< .0001			< .0001
Non-smoker	1,227,012 (54.2)	1,226,927 (54.2)	85 (52.8)		1,226,565 (54.2)	447 (50.28)		1,223,989 (54.16)	3023 (77.45)		1,226,724 (54.21)	288 (35.87)		1,217,815 (54.26)	9197 (47.34)	
Ex-smoker	404,394 (17.86)	404,357 (17.86)	37 (22.98)		404,088 (17.86)	306 (34.42)		403,957 (17.87)	437 (11.2)		404,197 (17.86)	197 (24.53)		400,092 (17.83)	4302 (22.14)	
Current smoker	632,447 (27.94)	632,408 (27.94)	39 (24.22)		632,311 (27.94)	136 (15.3)		632,004 (27.97)	443 (11.35)		632,129 (27.93)	318 (39.6)		626,518 (27.91)	5929 (30.52)	
Alcohol consumption, n (%)				0.0202			< .0001			< .0001			0.2546			0.0139
Never	1,234,060 (54.51)	1,233,958 (54.51)	102 (63.35)		1,233,468 (54.51)	592 (66.59)		1,230,759 (54.46)	3301 (84.58)		1,233,644 (54.51)	416 (51.81)		1,223,294 (54.5)	10,766 (55.41)	
Mild drinker	785,224 (34.69)	785,173 (34.69)	51 (31.68)		784,981 (34.69)	243 (27.33)		784,702 (34.72)	522 (13.37)		784,924 (34.68)	300 (37.36)		778,677 (34.69)	6547 (33.7)	
Heavy drinker	244,569 (10.8)	244,561 (10.8)	8 (4.97)		244,515 (10.81)	54 (6.07)		244,489 (10.82)	80 (2.05)		244,482 (10.8)	87 (10.83)		242,454 (10.8)	2115 (10.89)	
Regular physical activity, n (%)	469,505 (20.74)	469,475 (20.74)	30 (18.63)	0.5099	469,299 (20.74)	206 (23.17)	0.0735	468,799 (20.74)	706 (18.09)	< .0001	469,361 (20.74)	144 (17.93)	0.0498	465,297 (20.73)	4208 (21.66)	0.0015
Low income, n (%)	433,815 (19.16)	433,784 (19.16)	31 (19.25)	0.9763	433,673 (19.16)	142 (15.97)	0.0157	433,109 (19.16)	706 (18.09)	0.088	433,690 (19.16)	125 (15.57)	0.0096	429,979 (19.16)	3836 (19.74)	0.0384
Hypertension, n (%)	1,175,667 (51.93)	1,175,605 (51.93)	62 (38.51)	0.0007	1,175,288 (51.94)	379 (42.63)	< .0001	1,173,325 (51.92)	2342 (60.01)	< .0001	1,175,262 (51.93)	405 (50.44)	0.396	1,164,849 (51.9)	10,818 (55.68)	< .0001
Dyslipidemia, n (%)	851,217 (37.6)	851,178 (37.6)	39 (24.22)	0.0005	850,908 (37.6)	309 (34.76)	0.0801	849,590 (37.59)	1627 (41.69)	< .0001	850,912 (37.6)	305 (37.98)	0.823	842,921 (37.56)	8296 (42.7)	< .0001
Use of insulin, n (%)	167,639 (14.1)	167,616 (14.09)	23 (28.75)	0.0002	167,536 (14.09)	103 (20)	0.0001	167,021 (14.08)	618 (22.26)	< .0001	167,573 (14.1)	66 (15.64)	0.362	165,575 (14.06)	2064 (17.77)	< .0001
Number of oral hypoglycemic agents used ≥ 3, n (%)	299,387 (13.22)	299,371 (13.22)	16 (9.94)	0.2183	299,278 (13.23)	109 (12.26)	0.3962	298,801 (13.22)	586 (15.01)	0.001	299,295 (13.23)	92 (11.46)	0.1392	296,406 (13.21)	2981 (15.34)	< .0001
Duration of type 2 diabetes ≥ 5 years, n (%)	619,733 (27.38)	619,701 (27.38)	32 (19.88)	0.0328	619,482 (27.37)	251 (28.23)	0.5657	618,290 (27.36)	1443 (36.97)	< .0001	619,533 (27.38)	200 (24.91)	0.1166	613,809 (27.35)	5924 (30.49)	< .0001
Depression, n (%)	110,525 (4.88)	110,510 (4.88)	15 (9.32)	0.009	110,458 (4.88)	67 (7.54)	0.0002	110,089 (4.87)	436 (11.17)	< .0001	110,463 (4.88)	62 (7.72)	0.0002	109,197 (4.87)	1328 (6.84)	< .0001
Fasting glucose, mg/dL, Mean ± SD	138.46 ± 48.16	138.46 ± 48.16	128.45 ± 39.55	0.0083	138.47 ± 48.16	128.42 ± 41	< .0001	138.5 ± 48.16	118.96 ± 42.36	< .0001	138.46 ± 48.16	131.98 ± 51.63	0.0001	138.48 ± 48.16	136.25 ± 47.46	< .0001
Systolic BP, mmHg, Mean ± SD	128.73 ± 15.79	128.73 ± 15.79	123.47 ± 14.88	< .0001	128.73 ± 15.79	125.79 ± 14.97	< .0001	128.73 ± 15.79	128.94 ± 16.52	0.3558	128.73 ± 15.79	127.79 ± 15.3	0.0901	128.73 ± 15.79	128.55 ± 15.49	0.1062
Diastolic BP, mmHg, Mean ± SD	79.17 ± 10.27	79.17 ± 10.27	76.44 ± 9.14	0.0007	79.18 ± 10.27	77.3 ± 9.62	< .0001	79.18 ± 10.27	78.01 ± 10.1	< .0001	79.17 ± 10.27	79.23 ± 10.48	0.8801	79.18 ± 10.28	78.95 ± 10.12	0.0025
Total cholesterol, mg/dL, Mean ± SD	198.54 ± 46.39	198.54 ± 46.39	179.81 ± 42.65	< .0001	198.55 ± 46.39	189.24 ± 38.26	< .0001	198.55 ± 46.4	192.25 ± 40.64	0.0029	198.54 ± 46.39	191.87 ± 39.35	< .0001	198.54 ± 46.41	198.33 ± 44.31	0.5293
HDL cholesterol, mg/dL, Mean ± SD	51.44 ± 13.49	51.44 ± 13.49	49.71 ± 14.41	0.1028	51.44 ± 13.49	51.17 ± 13.59	0.5457	51.43 ± 13.48	55.64 ± 14.5	< .0001	51.44 ± 13.49	51.15 ± 13.68	0.537	51.44 ± 13.48	51.35 ± 13.58	0.3328
LDL cholesterol, mg/dL, Mean ± SD	113.81 ± 88.05	113.81 ± 88.05	98.3 ± 38.12	0.0254	113.81 ± 88.06	108.85 ± 34.34	0.0929	113.82 ± 88.1	109.38 ± 44	0.1495	113.81 ± 88.06	108.53 ± 34.9	0.0892	113.82 ± 88.3	113.03 ± 50.07	0.2148
Triglyceride, mg/dL, Median (IQR)	145.99 (145.88–146.1)	145.99 (145.88–146.1)	135.97 (124.79–148.16)	0.1164	146 (145.89–146.11)	124.6 (120.11–129.27)	< .0001	146.04 (145.93–146.14)	121.92 (120.08–123.8)	< .0001	145.99 (145.88–146.1)	137.81 (132.83–142.97)	0.0044	145.98 (145.87–146.09)	147.66 (146.51–148.82)	0.0054

*CD* crohn's disease, *UC* ulcerative colitis, *RA* rheumatoid arthritis, *AS* ankylosing spondylitis, *PsO* psoriasis, BMI body mass index, *BP* blood pressure, *HDL* high density lipoprotein, *LDL* low density lipoprotein, *SD* standard deviation, *IQR* interquartile range.

### Ethical approval and consent to participate

This study was approved by the Institutional Review Board (IRB) of Gangnam Severance Hospital (IRB No: 3-2020-0269). Owing to the retrospective nature of this study, the requirement for informed consent was waived.

## Results

### Baseline characteristics

The baseline characteristics of the 2,263,853 patients with type 2 diabetes, and the comparisons of their characteristics according to each IMID are summarized in Table 1. Of the 2,263,853 patients with type 2 diabetes, 161 (0.007%), 889 (0.039%), 3903 (0.159%), 803 (0.035%), and 19,428 (0.858%) patients had CD, UC, RA, AS, and PsO, respectively. The mean age of the study population was 55.65 ± 12.31 years, and 1,388,001 (61.31%) patients were male.

The proportion of males as well as the mean age differed between groups of patients with each IMID. In comparisons with the respective controls, in those with CD and those with AS, males and younger age were commoner; in those with UC and those with PsO, males and older age were commoner; and in those with RA, females and older age were commoner. According to each IMID, the proportions of current smokers, heavy alcohol drinkers, and those who perform regular physical activity varied. In comparisons with their respective controls, those with CD included a lower proportion of heavy alcohol drinkers; those with UC included a lower proportion of current smokers and heavy alcohol drinkers; those with RA included a lower proportion of current smokers, heavy alcohol drinkers, and those who perform regular physical activity; those with AS included a higher proportion of current smokers and lower proportion of patients who perform regular physical activity; and those with PsO included a higher proportion of current smokers and those who perform regular physical activity. The presence of other cardiovascular risk factors varied between groups of patients with each IMID as well. Hypertension and dyslipidemia were less common in patients with CD; hypertension was less common in patients with UC; and hypertension and dyslipidemia were commoner in patients with RA and those with PsO, in comparisons with their respective controls.

### Incidence of cardiovascular mortality according to IMIDs

**Figure 2:**
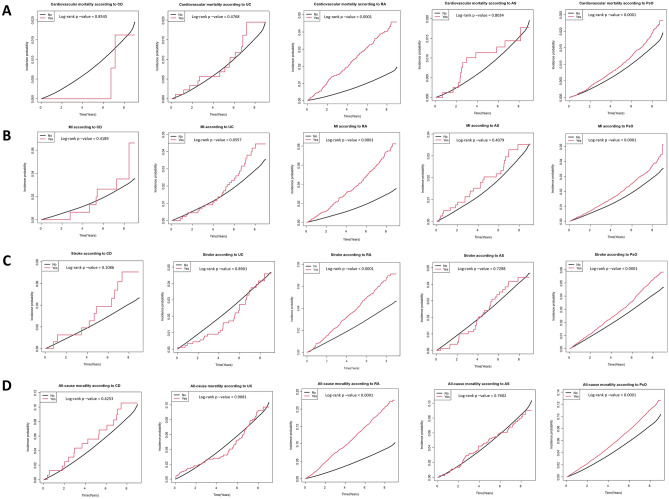
Comparison of cumulative incidence of (**A**) cardiovascular mortality, (**B**) MI, (**C**), stroke, and (**D**) all-cause mortality according to the presence of each IMID. CD, crohn’s disease; UC, ulcerative colitis; RA, rheumatoid arthritis; AS, ankylosing spondylitis; PsO, psoriasis; MI, myocardial infarction; IMIDs, immune-mediated inflammatory disease.

**Figure 3:**
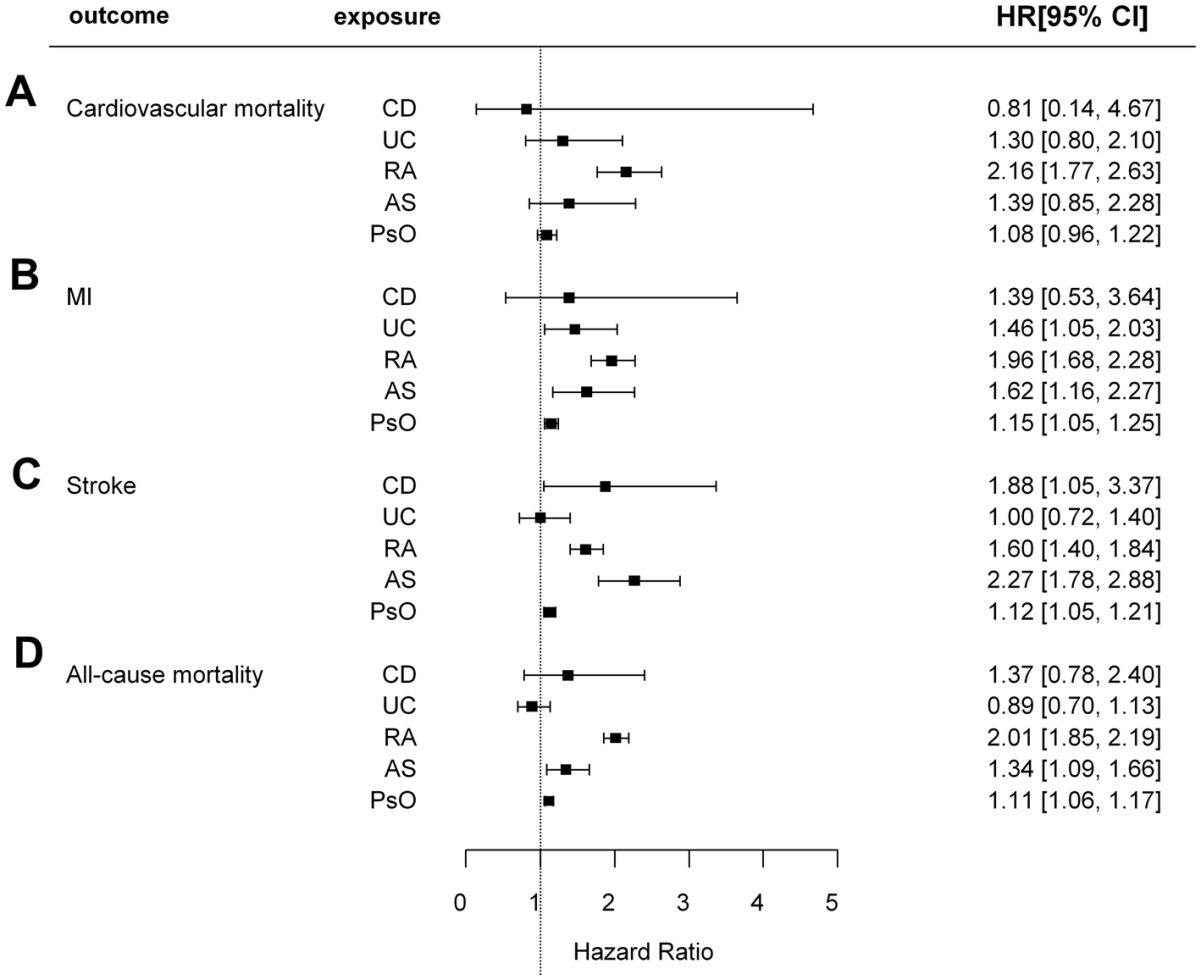
A forest plot showing IPTW-adjusted HRs and 95% CIs for (**A**) cardiovascular mortality, (**B**) MI, (**C**), stroke, and (**D**) all-cause mortality. IPTW, inverse probability of treatment weighting; HR, hazard ratio; CI, confidence interval; CD, Crohn’s disease; UC, ulcerative colitis; RA, rheumatoid arthritis; AS, ankylosing spondylitis; PsO, psoriasis; MI, myocardial infarction.

The incidences of cardiovascular mortality in patients with type 2 diabetes with CD, UC, RA, AS, and PsO were 1.67, 2.28, 8.56, 2.04, and 2.40 per 1000 person-years, respectively. The cumulative incidence of cardiovascular mortality according to the presence of each IMID is depicted in Fig. 2A. Patients with RA (*p* < 0.001) and PsO (*p* < 0.001) had a significantly higher cumulative incidence of cardiovascular mortality than those without RA and PsO, respectively. Compared with their respective controls, patients with RA (unadjusted HR: 2.855 [95% CI 2.421, 3.367]) and PsO (unadjusted HR: 1.273 [95% CI 1.144, 1.418]) had a significantly higher risk of cardiovascular mortality in the univariable model (model 1). The higher risk of cardiovascular mortality remained statistically significant only in patients with RA in the multivariable models (model 2–6) that were adjusted for potential confounders (model 6, adjusted HR: 2.278 [95% CI 1.931, 2.689]). Similarly, IPTW-adjusted analysis revealed higher risk of cardiovascular mortality in patients with RA (IPTW-adjusted HR: 2.156 [95% CI 1.769, 2.627]). There was no difference in the cardiovascular mortality between patients with type 2 diabetes based on the presence of CD, UC, and AS, respectively (Table 2 and Fig. 3).

**Table 2:** Clinical outcomes according to the presence of immune-mediated inflammatory diseases.

Clinical outcomes	Disease group		Control group		HR (95% CI)						
	No. of events/N	Incidence rate (/1000 person-years)	No. of events/N	Incidence rate (/1000 person-years)	Model 1	Model 2	Model 3	Model 4	Model 5	Model 6	IPTW-adjusted
**Cardiovascular mortality**											
CD	2/161	1.67478	31,394/2,263,692	1.89188	0.886 (0.223,3.521)	1.162 (0.290,4.645)	1.194 (0.299,4.775)	1.096 (0.274,4.384)	1.543 (0.386,6.169)	1.085 (0.271,4.337)	0.812 (0.141,4.674)
UC	15/889	2.28232	31,381/2,262,964	1.89171	1.202 (0.724,1.993)	1.054 (0.636,1.749)	1.084 (0.653,1.798)	1.105 (0.666,1.834)	0.933 (0.502,1.734)	1.104 (0.665,1.831)	1.298 (0.803,2.100)
RA	142/3903	8.55730	31,254/2,259,950	1.88634	2.855 (2.421,3.367)	2.471 (2.095,2.914)	2.452 (2.079,2.892)	2.270 (1.925,2.678)	2.263 (1.884,2.718)	2.278 (1.931,2.689)	2.156 (1.769,2.627)
AS	12/803	2.03583	31,384/2,263,050	1.89181	1.077 (0.612,1.896)	1.663 (0.945,2.929)	1.623 (0.922,2.858)	1.592 (0.904,2.804)	1.195 (0.537,2.660)	1.632 (0.926,2.873)	1.390 (0.847,2.282)
PsO	336/19,428	2.39507	31,060/2,244,425	1.88757	1.273 (1.144,1.418)	1.093 (0.981,1.217)	1.079 (0.969,1.202)	1.077 (0.967,1.199)	1.029 (0.905,1.169)	1.051 (0.944,1.170)	1.081 (0.960,1.218)
**MI**											
CD	6/161	5.07558	59,656/2,263,692	3.63053	1.391 (0.626,3.095)	1.633 (0.735,3.629)	1.635 (0.735,3.634)	1.654 (0.743,3.681)	1.599 (0.600,4.261)	1.608 (0.722,3.579)	1.390 (0.530,3.643)
UC	33/889	5.07397	59,629/2,262,964	3.63006	1.396 (0.993,1.963)	1.285 (0.913,1.807)	1.302 (0.926,1.831)	1.327 (0.943,1.867)	1.149 (0.749,1.762)	1.332 (0.946,1.873)	1.462 (1.051,2.032)
RA	237/3903	9.03496	59,425/2,259,950	3.62199	2.532 (2.228,2.876)	2.224 (1.957,2.527)	2.165 (1.906,2.460)	2.152 (1.894,2.445)	1.940 (1.675,2.247)	2.154 (1.895,2.447)	1.958 (1.683,2.278)
AS	25/803	4.29056	59,637/2,263,050	3.63040	1.197 (0.811,1.767)	1.477 (0.998,2.186)	1.419 (0.959,2.101)	1.400 (0.946,2.072)	1.748 (1.140,2.681)	1.402 (0.947,2.075)	1.624 (1.164,2.266)
PsO	648/19,428	4.67600	59,014/2,244,425	3.62174	1.296 (1.200,1.400)	1.181 (1.093,1.276)	1.158 (1.071,1.251)	1.143 (1.058,1.235)	1.075 (0.979,1.180)	1.115 (1.032,1.205)	1.146 (1.055,1.246)
**Stroke**											
CD	10/161	8.59329	84,853/2,263,692	5.19290	1.670 (0.901,3.094)	2.046 (1.101,3.802)	2.140 (1.151,3.977)	2.105 (1.133,3.912)	2.837 (1.482,5.431)	1.814 (1.074,3.063)	1.877 (1.046,3.367)
UC	33/889	5.07569	84,830/2,262,964	5.19319	0.977 (0.694,1.374)	0.886 (0.630,1.246)	0.928 (0.660,1.306)	0.949 (0.674,1.335)	0.914 (0.612,1.363)	1.085 (0.844,1.395)	1.000 (0.717,1.395)
RA	224/3903	8.55730	84,639/2,259,950	5.18775	1.657 (1.453,1.889)	1.405 (1.232,1.602)	1.406 (1.233,1.603)	1.382 (1.212,1.576)	1.289 (1.111,1.496)	1.702 (1.547,1.871)	1.605 (1.396,1.845)
AS	32/803	5.51876	84,831/2,263,050	5.19303	1.065 (0.753,1.506)	1.448 (1.024,2.047)	1.433 (1.013,2.027)	1.412 (0.998,1.996)	1.183 (0.754,1.854)	1.420 (1.087,1.854)	2.266 (1.782,2.882)
PsO	913/19,428	6.63109	83,950/2,244,425	5.18093	1.282 (1.201,1.368)	1.146 (1.074,1.223)	1.139 (1.068,1.216)	1.135 (1.064,1.212)	1.110 (1.028,1.199)	1.107 (1.052,1.166)	1.123 (1.046,1.205)
**All-cause mortality**											
CD	16/161	13.3982	181,262/2,263,692	10.9233	1.246 (0.767,2.024)	1.572 (0.963,2.566)	1.623 (0.994,2.650)	1.465 (0.897,2.391)	1.436 (0.795,2.593)	1.428 (0.875,2.330)	1.372 (0.784,2.403)
UC	73/889	11.1073	181,205/2,262,964	10.9234	1.000 (0.794,1.260)	0.868 (0.690,1.092)	0.898 (0.714,1.129)	0.893 (0.710,1.124)	0.849 (0.654,1.104)	0.857 (0.682,1.079)	0.887 (0.696,1.130)
RA	750/3903	28.0406	180,528/2,259,950	10.8958	2.600 (2.421,2.794)	2.439 (2.271,2.621)	2.424 (2.256,2.604)	2.207 (2.054,2.371)	2.021 (1.863,2.193)	2.130 (1.982,2.289)	2.013 (1.849,2.192)
AS	62/803	10.5184	181,216/2,263,050	10.9236	0.963 (0.751,1.235)	1.355 (1.057,1.739)	1.331 (1.038,1.707)	1.311 (1.022,1.682)	1.258 (0.923,1.715)	1.324 (1.032,1.699)	1.344 (1.089,1.658)
PsO	2025/19,428	14.4346	179,253/2,244,425	10.8935	1.329 (1.272,1.389)	1.117 (1.069,1.167)	1.105 (1.057,1.154)	1.117 (1.069,1.167)	1.056 (1.003,1.112)	1.080 (1.034,1.129)	1.115 (1.062,1.171)

*HR* hazard ratio, *CI* confidence interval, *CD* Crohn's disease, *UC* ulcerative colitis, *RA* rheumatoid arthritis, *AS* ankylosing spondylitis, *PsO* psoriasis, *MI* myocardial infarction, *IPTW* inverse probability of treatment weighting.

Model 1 adjusted for none of the covariates (univariable analysis).

Model 2 adjusted for age and sex.

Model 3 adjusted for age, sex, smoking, alcohol consumption, and regular physical activity.

Model 4 adjusted for age, sex, smoking, alcohol consumption, regular physical activity, hypertension, dyslipidemia, and BMI.

Model 5 adjusted for age, sex, smoking, alcohol consumption, regular physical activity, hypertension, dyslipidemia, BMI, use of insulin, number of oral hypoglycemic agent ≥ 3, duration of type 2 diabetes ≥ 5 years, and depression.

Model 6 adjusted for age, sex, smoking, alcohol consumption, regular physical activity, hypertension, dyslipidemia, BMI, use of insulin, number of oral hypoglycemic agent ≥ 3, duration of type 2 diabetes ≥ 5 years, depression, waist circumference, fasting glucose, systolic BP, diastolic BP, total cholesterol, LDL-cholesterol, and triglyceride.

### Incidence of MI and stroke, and all-cause mortality rate according to IMIDs

The incidences of MI in patients with type 2 diabetes with CD, UC, RA, AS, and PsO were 5.08, 5.07, 9.03, 4.29, and 4.68 per 1000 person-years, respectively. The cumulative incidence of MI according to each IMID is depicted in Fig. 2B. Patients with RA (*p* < 0.001) and PsO (*p* < 0.001) had a significantly higher cumulative incidence of MI than those without RA and PsO, respectively. In the univariable model (model 1), patients with RA (unadjusted HR: 2.532 [95% CI 2.228, 2.876]) and those with PsO (unadjusted HR: 1.296 [95% CI 1.200, 1.400]) had significantly higher risks of MI. After adjusting for the potential confounders, patients with RA continued to demonstrate significantly higher risk of MI (model 6, adjusted HR: 2.154 [95% CI 1.895, 2.447]), as well as the patients with PsO (model 6, adjusted HR: 1.115 [95% CI 1.032, 1.205]). These associations were also observed in the IPTW-adjusted analysis: higher risk of MI in patients with RA (IPTW-adjusted HR: 1.958 [95% CI 1.683, 2.278]), and those with PsO (IPTW-adjusted HR: 1.146 [95% CI 1.053, 1.246]). Moreover, patients with UC (IPTW-adjusted HR: 1.462 [95% CI 1.051, 2.032]) and those with AS (IPTW-adjusted HR: 1.624 [95% CI 1.164, 2.266]) were newly identified to have a significantly higher risk of MI in the IPTW-adjusted analysis. There were no differences in the risk of MI in patients with type 2 diabetes with CD (Table 2 and Fig. 3).

The incidences of stroke in patients with type 2 diabetes with CD, UC, RA, AS, and PsO were 8.59, 5.08, 8.56, 5.52, and 6.63 per 1000 person-years, respectively. The cumulative incidence of stroke according to each IMID is depicted in Fig. 2C. Patients with RA (*p* < 0.001) and PsO (*p* < 0.001) had a significantly higher cumulative incidence of stroke than those without RA and PsO, respectively. Patients with RA (unadjusted HR: 1.657 [95% CI 1.453, 1.889]) and PsO (unadjusted HR: 1.282 [95% CI 1.201, 1.368]) had a significantly higher risk of stroke in the univariable model (model 1) compared with their respective controls. The higher risk of stroke remained statistically significant in the multivariable models in patients with RA (model 6, adjusted HR: 1.702 [95% CI 1.547, 1.871]) and those with PsO (model 6, adjusted HR: 1.107 [95% CI 1.052, 1.166]). Patients with type 2 diabetes with CD (adjusted HR: 1.814 [95% CI 1.074, 3.063]) and those with AS (adjusted HR: 1.420 [95% CI 1.087, 1.854]) were newly identified to have a significantly higher risk of stroke in model 6. Similar associations were also observed in the IPTW-adjusted analysis: higher risk of stroke in patients with CD (IPTW-adjusted HR: 1.877 [95% CI 1.046, 3.367]), those with RA (IPTW-adjusted HR: 1.605 [95% CI 1.396, 1.845]), those with AS (IPTW-adjusted HR: 2.266 [95% CI 1.782, 2.882]), and those with PsO (IPTW-adjusted HR: 1.123 [95% CI 1.046, 1.205]). There was no difference in the risk of stroke in patients with type 2 diabetes with UC (Table 2 and Fig. 3).

The rates of all-cause mortality in patients with type 2 diabetes with CD, UC, RA, AS, and PsO were 13.40, 11.11, 28.04, 10.52, and 14.43 per 1000 person-years, respectively. The cumulative incidence of all-cause mortality according to each IMID is depicted in Fig. 2D. Patients with RA (*p* < 0.001) and PsO (*p* < 0.001) had a significantly higher cumulative incidence of all-cause mortality than those without RA and PsO, respectively. In the univariable model (model 1), patients with RA (unadjusted HR: 2.600 [95% CI 2.421, 2.794]) and PsO (unadjusted HR: 1.329 [95% CI 1.272, 1.389]) had a significantly higher risk of all-cause mortality—both these risk remained statistically significant in all the multivariable models (model 6, RA, adjusted HR: 2.130 [95% CI 1.982, 2.289]; and model 6, PsO, adjusted HR: 1.080 [95% CI 1.034, 1.129]). Patients with type 2 diabetes with AS (adjusted HR: 1.324 [95% CI 1.032, 1.699]) were newly identified to have a significantly higher risk of all-cause mortality in model 6. Similar associations were also observed in the IPTW-adjusted analysis: higher risk of all-cause mortality in patients with RA (IPTW-adjusted HR: 2.013 [95% CI 1.849, 2.192]), those with AS (IPTW-adjusted HR: 1.344 [95% CI 1.089, 1.658]), and those with PsO (IPTW-adjusted HR: 1.115 [95% CI 1.062, 1.171]). There were no differences in the rates of all-cause mortality between patients with type 2 diabetes with CD, and UC (Table 2 and Fig. 3).

## Discussion

In this population-based cohort study, we found significant associations between the presence of IMIDs and the risks of cardiovascular morbidity and mortality in patients with type 2 diabetes. Notably, the effects on cardiovascular morbidity and mortality varied between IMIDs. CD was associated with a higher risk of stroke; UC was associated with a higher risk of MI; RA was associated with a higher risk of cardiovascular mortality, MI, stroke, and all-cause mortality; AS was associated with a higher risk of MI, stroke, and all-cause mortality; and PsO was associated with a higher risk of MI, stroke, and all-cause mortality. These findings were observed in multivariable models adjusted for potential confounders, such as the traditional cardiovascular risk factors (age, sex, hypertension, dyslipidemia, smoking, and obesity)^25^, as well as in the IPTW-adjusted analyses, thus suggesting that IMIDs should be considered independent risk factors of cardiovascular disease in patients with type 2 diabetes. To our knowledge, this is the first study to comprehensively evaluate the effects of IMIDs on cardiovascular morbidity and mortality in patients with type 2 diabetes. The association between each and every IMID with risk of cardiovascular morbidity and mortality observed in our study reflects that chronic inflammation is an important cardiovascular risk factor. Considering that cardiovascular diseases cause substantial economic burden and adversely affect the quality of life in patients with type 2 diabetes, patients with concomitant IMIDs definitely deserve more stringent monitoring for cardiovascular morbidity and mortality.

Among the IMIDs, RA was the most potent risk factor of cardiovascular mortality, MI, and all-cause mortality with approximately a two-fold increase in the risk, while AS was the most potent risk factor of stroke with approximately a two-fold increase in the risk. The effect sizes of these diseases on the cardiovascular outcomes were larger than that observed in the general population^15,26–28^. Previous studies have reported increased risk of MI by approximately 68% and that of cerebrovascular accidents by approximately 41% in patients with RA when compared with the general population^26–28^. In addition, a meta-analysis reported that AS was associated with a 50% increased risk of stroke^15^. Given that type 2 diabetes itself is associated with an increased risk of cardiovascular morbidity and mortality^2–5^, the effect sizes of RA and AS on the cardiovascular outcomes observed in this study are considerable. Hence, patients with type 2 diabetes who also have RA or AS could benefit from a more vigilant surveillance for cardiovascular diseases and a tighter control of modifiable cardiovascular risk factors.

In regard to IBD, CD, but not UC, demonstrated a significant association with stroke. Similarly, a recent study that assessed the risk of stroke in patients with IBD reported a higher risk of stroke in CD (HR: 1.50 [95% CI 1.10, 2.06]) but not in UC (HR: 1.17 [95% CI 0.90, 1.52]) when compared with those without IBD^29^. Additionally, a meta-analysis of articles published before July 2020 demonstrated that CD was associated with a 25% increased risk of stroke^30^. Inflammation is a major contributor to accelerated atherosclerosis, which consequently results in cardiovascular morbidities^31,32^. Considering that UC is less aggressive and has less frequent extra-intestinal manifestations than CD^33^, the lower inflammatory burden of UC than that in CD could be a possible explanation for the lack of association between UC and stroke. Regarding other outcomes, UC was associated with a higher risk of MI. This is similar to the findings of a previous study in the general population that demonstrated a higher risk of MI in patients with IBD (OR: 1.25 [95% CI 1.24, 1.27])^13^. However, in our study, CD was not associated with a higher risk of MI. It should be noted that in our study, the HRs of CD had wide 95% CIs, probably due to a small number of patients (n = 161). The effect size of CD on MI (IPTW-adjusted: 1.390) was numerically comparable to that of UC on MI (IPTW-adjusted HR: 1.462), and even higher than that reported in the previous study (OR 1.25)^13^. Therefore, although the statistical significance was not reached in the association between CD and MI, patients with CD might still benefit clinically from stringent monitoring for MI.

Regarding AS, a meta-analysis reported a higher risk of MI (OR 1.60 [95% CI 1.32, 1.93]) and stroke (OR 1.50 [95% CI 1.39, 1.62]) in patients with AS in the general population^15^. Another study demonstrated a significantly higher risk of cardiovascular mortality (HR 1.36 [95% CI 1.13, 1.65]) in patients with AS^34^. We also observed a significantly higher risk of MI (IPTW-adjusted HR: 1.624 [95% CI 1.164, 2.266]) and stroke (IPTW-adjusted HR 2.266 [95% CI 1.782, 2.882]) in patients with type 2 diabetes with a larger effect size than that observed in the general population^15^. Moreover, associations of AS with all cause-mortality was also observed in this study. This might be explained by the relatively older age of patients with type 2 diabetes and AS. The mean age of the patients with type 2 diabetes and AS in our study was 50.05 years, whereas it was 45.55 years in the previous study^34^. The older age of patients with AS in our cohort may have accentuated the effect of AS on MI, stroke, and all-cause mortality.

PsO has been reported to increase the risk of MI (OR 1.25)^16^. In our study, PsO was associated with only a modestly higher risk of MI (IPTW-adjusted HR 1.146 [95% CI 1.055, 1.246]). Furthermore, PsO was also associated with a modestly higher risk of stroke and all-cause mortality (stroke, IPTW-adjusted HR: 1.123 [95% CI 1.046, 1.205]; all-cause mortality, IPTW-adjusted HR: 1.115 [95% CI 1.062, 1.171]). A population-based study that investigated the epidemiology of PsO in Korea reported that mild PsO is 7–9 times commoner than severe PsO^23^. It is possible that the low proportion of patients with severe PsO in the Korean population may have led to the smaller effect size. Therefore, although the effect size was relatively small compared with those of other IMIDs, PsO deserves close attention in patients with type 2 diabetes.

There are some limitations to this study. First, this was a retrospective observational study. Although we adjusted for a number of potential confounders in the multivariable models, the possibility of confounding by unmeasured covariates exists. For instance, data on the use of medications for IMIDs and the disease activities of these IMIDs, which are potential confounders for cardiovascular outcomes^25^, were not available. In addition, hemoglobin A1c levels were also not available. Second, as this was a Korean population-based study, the results may not be generalizable to other populations. Nonetheless, this study has a strength in that a large cohort of patients with type 2 diabetes extracted from the nationwide population database was used. This enabled us to evaluate the effects of IMIDs, which are relatively rare diseases, on the cardiovascular outcomes in patients with type 2 diabetes.

## Conclusions

**Figure 4:**
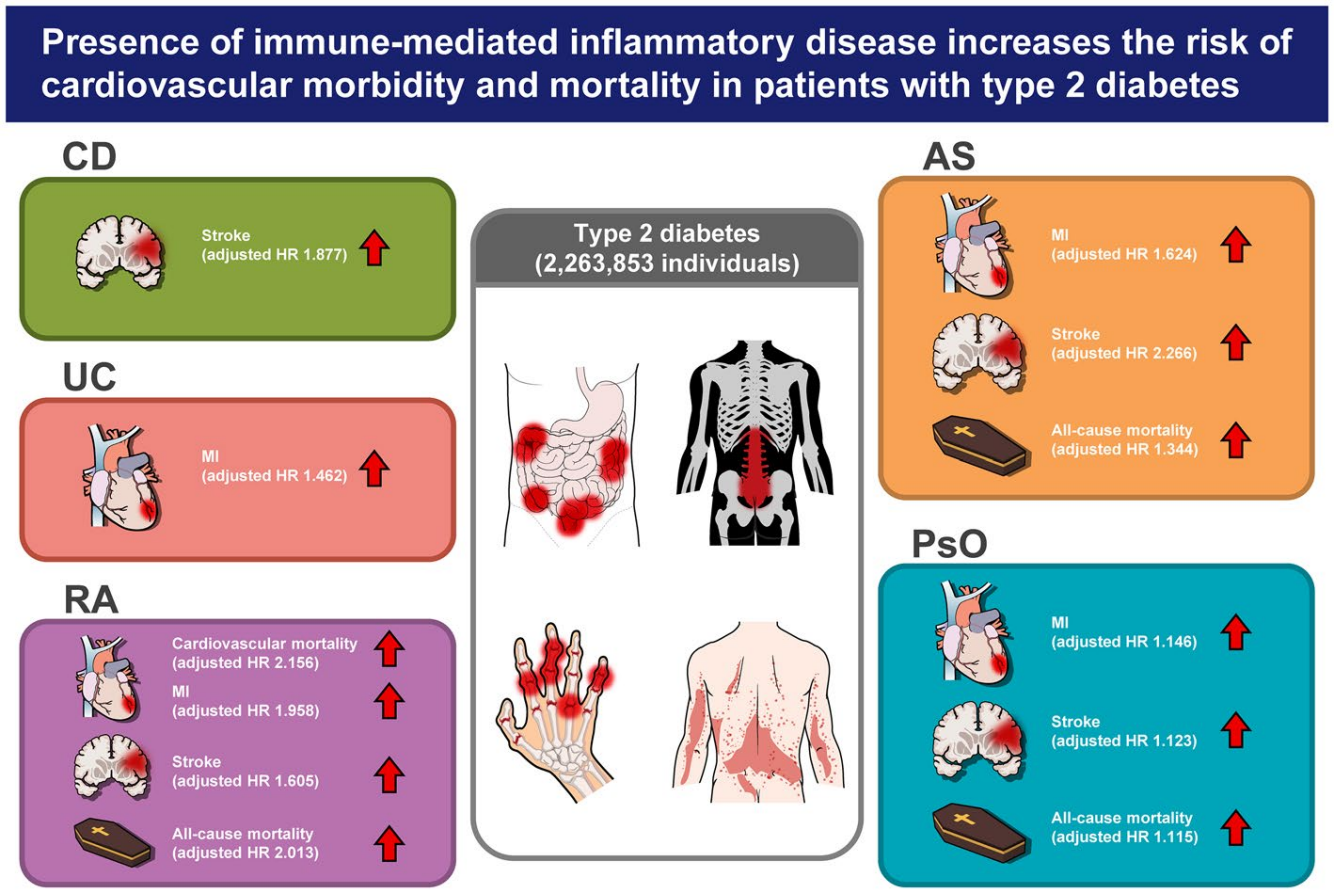
Summary of the present nationwide population-based study. CD, crohn’s disease; UC, ulcerative colitis; RA, rheumatoid arthritis; AS, ankylosing spondylitis; PsO, psoriasis; HR, hazard ratio.

In conclusion, the presence of IMIDs in patients with type 2 diabetes was associated with a higher risk of cardiovascular morbidity and mortality (Fig. 4). Particularly CD was associated with a higher risk of stroke; UC was associated with a higher risk of MI; RA was associated with a higher risk of cardiovascular mortality, MI, stroke, and all-cause mortality; AS was associated with a higher risk of MI, stroke, and all-cause mortality; and PsO was associated with a higher risk of MI, stroke, and all-cause mortality. Therefore, patients with type 2 diabetes with concomitant IMIDs require meticulous surveillance for cardiovascular morbidity and mortality.

## Data Availability

All data generated or analyzed during this study are included in this article.

## Abbreviations

IMIDs: Immune-mediated inflammatory diseases
CD: Crohn’s disease
UC: Ulcerative colitis
RA: Rheumatoid arthritis
AS: Ankylosing spondylitis
PsO: Psoriasis
IBD: Inflammatory bowel disease
MI: Myocardial infarction
OR: Odds ratio
NHIS: National Health Insurance Service
RID: Rare intractable disease
IRB: Institutional review board
HR: Hazard ratio
CI: Confidence interval
